# Is Woman Adapted to the Dental Profession?

**Published:** 1873-04

**Authors:** Emilie Foeking

**Affiliations:** Dantzig, Prussia.


### ARTICLE II.

### *Is Woman Adapted to the Dental Profession?*

BY MISS EMILIE FOEKING, D. D. S., DANTZIG, PRUSSIA.

At the opening of this paper I beg to be allowed the remark, that I do not rank—nor do I wish to rank—among that class of women commonly called the "strong minded," and hope that all who have known me during my two courses of study in the Baltimore College of Dental Surgery will kindly bear witness to this statement of mine.

I will merely try to prove in this manuscript that woman is able to pursue the study of dentistry, without being in any way suspected of thus attempting to attain to an aim lying far beyond the reach of her power, or being accused of meddling with the sphere of men.

Being a foreigner, I confidently hope that you will kindly bear with my English, and be lenient critics, if a deficient knowledge of the language occasionally prevents me from giving the precise and clear idea of what I wish to say.

We often hear women accused of trespassing on the realms of men by overstepping the limits drawn for them by nature, custom and law. It is true, the Bible seems but little in favor of rights being conferred upon women, for in Genesis, 4th chapter, 10th verse, we read that the Lord says to Eve, "Man shall rule over thee." The same discouraging and humiliating view we again meet with in the New Testament. 1st Corinthians, 14th chapter, 34th verse, we read, "Let your women keep silence in the churches; for it is not permitted unto them to speak, but they are commanded to be under obedience."

Infallibility long claimed for the Holy Scriptures, and since the 18th of July, 1870, even for one single man, cannot be defended any longer in the strict meaning by the church now-a-days. We do not study any more natural history from the Bible, nor do we approve of what, in the name of God, has been done and greatly favored by the church in by-gone days, or brought forth what formerly constituted a favorite occupation of saints, so-called.

The burning of witches on the pyre, and putting to death heretics, to the greatest grief of a certain class of people, have been abandoned, never to take place again; and nobody—even the most fanatical zealot—needs to be sorry for that. Contrary to the words given above, "as saith the law," women are the members of church most looked for now; the pews are filled with the fair sex; and on church fairs, when the question of raising money comes up, women are certainly an aid much desired.

The light diffused by science in the course of time has driven the obscurants to their dens, there to bewail the loss of the "good old times."

But then the very same church has also acknowledged the equality of women by making women saints—thus one for not washing her face a lustrum or more, and others upon grounds of similar significance. Nay, we are told even that a woman by the name of Joanas VIII, occupied the Papal chair, who, unluckily to her, had to pay the frailty of her sex. In this act, (provided it be true,) the wrong done to women by the Bible is amply repaid by the church.

Civil law, from period to period, has been changed in favor of women, and none of them need to complain in our days of not getting their rights before any civilized court, even if not assisted by man—which formerly was a necessary condition. Civil law, as the exponent of the sentiments and feelings of the time in which it arose, as far as woman's position is concerned, shows a gradual change for the better. In mediæval times a man was fined six solidi for killing a serf-woman, and six hundred for killing a lady. Now-a-days equal punishment is dealt out to the murderer, no matter whether he kills a poor negro woman or a queen.

We all very well know that social customs are not the product of one day. They change slowly—so slowly indeed that the observer is hardly able to notice the change, except he be a close observer and outlive several generations. Social customs are quite the contrary to political party ideas, which may be formed within twenty-four hours, eke out a short existence, and die off as quickly as they have sprouted up. In the course of time many prejudices have been, and will yet be given up. And if truth steadfastly refuses to strike her topsails in compliment to ignorance and prejudice, she will reach her aim all the earlier.

This country, comparatively free from prejudice, and unencumbered by the drones of nobility-by-birth, has extended more chances to thriving women than any other on the globe. Is this country not educated by an army of women employed among us as teachers? Offices in the civil service have been entrusted to women, and so far as I hear, to the fullest satisfaction of the government, as well as great credit to themselves. Now in former days it was not the custom to select women as school teachers; thus one had to innovate the change. It was not deemed suitable or proper for a lady to hold a position in a government office; thus one had to introduce the new order of things. These innovators, of course, were accused of being intruders upon the rights of men; they were considered unwomanly.

Now as regards the charge brought against women, that in their claim for certain rights they were going beyond the limits fixed for them by nature, I will but briefly remark, that a man afflicted with the stitch has not yet been seen running a race.

I well know that the command of the Lord, given to the first man, (Genesis, 1st ch., 18th v.,) still holds good for our days. But as long as gynecocracy shall not reach that point which enables her to compel a man she likes to marry her, so long we will have unmarried women. And such, even if they are rich, lead a tedious life at the best as spinsters; and if they are poor, will either become an incumbrance to their family, or submit themselves to any work to which they, perhaps, have not been used in their earlier days. At all hazards, they are generally considered of but little use in the world, because they have no aim to strive for; they have nothing, even if fate deals well with them, but their work-box at their elbow for consolation. However, to be aimless means to be useless. Therein lies the reason why old maidens almost everywhere are objects of ridicule—which is not the case with old bachelors. They carry on their business, or follow some profession, and are apparently of some benefit to the world in one way or another. Hence, if woman abandons the old and worn-out highroad of social custom, of bitter disappointment, humiliation and distress, by engaging in some trade, business or profession congenial and suitable to her inclination, talents and physical strength, should she on this account be blamed and reproached? Surely not all females can become good governesses—for, heaven knows, we have too many of them already, frightening children with their ogre-eyed spectacles of various colors, and their restlessly and nervously quivering curls and lips. Moreover, their lot being by no means an enviable one, I concluded not to increase their number, but to engage in the practice of dentistry. I shall endeavor to prove not only the right of woman to apply herself to such a study, but also her indubitable qualification, superior often to that of man in the practice of this profession.

A.—*What are the Objects of Dentistry?*

The offices of this profession are three—

First: The treatment necessary for the preservation of healthy teeth.

Second: The best treatment for the cure of diseased teeth, and their removal when this may become necessary.

Third: The replacement of the teeth removed by artificial ones.

B.—*Who can Perform these Duties?*

After speaking of the aim and scope of dentistry, the question as to the necessary qualification for the study and practice of this art presents itself for consideration.

It is true, almost any one may pull a loose incisor; and there are, moreover, enough men and women who carry on this business of tooth-pulling. However, as an usual thing, these persons care but little if, by their unskilled and ignorant manipulations, they injure those entrusting themselves to such unprofessional hands. I saw a case of this kind in Germany. A blacksmith's wife, who formerly had been a servant in a barber's house, and there had often witnessed teeth pulling, took a fancy to it and practiced it. In a country village, where I happened to be at the time, she tried to pull a molar, but after seven unsuccessful attempts she finally fractured the jaw-bone of the unfortunate sufferer. Unskilled hands, no doubt, will often do more harm than good, and should keep from meddling with this art.

But where is this skill to be obtained? Surely nowhere better than in dental colleges. Dentistry being taught theoretically by lectures, and practically by demonstrative instruction in operating, the first qualification indispensable to those wishing to enter upon this study will be, that such persons should be in the possession of an education sufficient to enable them to follow up with profit the lectures of the professors. If the student has obtained a classical education it is so much the better—for it will greatly assist him or her in the preliminary studies of this art and science. I feel myself only too painfully how hard it is to understand and retain the technical terms in use without a sufficient knowledge of the Latin language. However, one may possess the necessary educational foundation and yet be wholly unfit practically to pursue this profession; for there are many who cannot endure the sight of blood, and yet bloody work has often to be done in dentistry. Strong nerves, calmness and courage are indispensable for the pursuit of this profession. Moreover, since many of the operations pertaining to dentistry require a certain amount of physical strength, the practitioner of this art must necessarily be in possession of it, or many of his attempts in practical dentistry will result in failures.

Not only physical strength, but also mechanical and artistical skill is required to satisfy the demands duly made upon practical dentists. A person may have the strength of a lion, yet, having five thumbs on each hand, will surely be little qualified to do justice to this work, and must give poor satisfaction to his patrons.

C.—*Can a Woman Perform these Duties?*

It now involves upon me to answer the question, whether woman possesses the qualifications necessary for the study and practice of dentistry. Surely not all women are able successfully to engage in such a study; but this holds good with regard to man. The latter, it is true, has many advantages over the former. Our high schools and colleges, offering him a classical education, are open to him, so that in this respect there is no obstacle in his way that prevents him from preparing himself for any profession.

In Germany, on the other hand, it is difficult for women to obtain a classical and scientific training, attention being paid there mostly to the study of French and English, and to the perusal of our own and foreign literature. Neither chemistry, anatomy or physiology is studied there by women, and what little is done in natural sciences is hardly worth speaking of. Latin is not taught to females in any of our institutions—at least not so far as I know of. Prussia has 407 high schools, (universities, with about 7,500 students, not included,) for boys and young men, with 140,000 pupils, but I do not know of any such institution for the benefit of girls, which enjoys the support of the government. The whole care of providing for female schools is left either to private enterprise or to the various cities. Prussia expends from thirty to forty-five per cent. of her whole income for military purposes, and not quite three per cent. for schools. If, therefore, a woman desires to enter upon any professional duty, she must privately undergo that preliminary training which qualifies her successfully to pursue the former.

There is, however, another point worthy of consideration. Girls leaving school at the age of seventeen or eighteen very rarely think of undertaking the study of medicine or any other profession. At least the number will be an exceedingly small one in Germany, for at that age almost all expect to marry. However, many a worthy young girl sees her hopes blasted in this respect, and finding herself doomed to travel through life alone, feels the necessity of providing for her own wants. Thus, when the hand of disappointed and advanced womanhood gradually but mercilessly begins to destroy her youthful charms, and marks the number of her lonely hours, and the blighted hopes on her thoughtful brow, she begins to look around for some honorable means of self-support, and to think of some professional pursuit. But much of what she has learned in school has been forgotten, and now has to be learned over again. This requires no little perseverance, and is accomplished with no little strain upon both mind and body. However, this very perseverance is the best argument in favor of the right of woman to shape her own destiny, and to choose her own path of life, if fate shows itself unwilling to allow her to reach the aim which nature desires every woman to reach for her own happiness as well as the happiness of others. And whenever we meet with such perseverance we should not ruin that courage which arms one to come up to one's duty with strong nerves and steady hands. Whoever of women has such a mind, she need not be afraid "to put the hand to the plough."

It is true the work to be performed, the labor to be done, and hardships to be undergone, is great—certainly greater than I had imagined. Indeed we often entertain wrong ideas about matters we do not know; but at the same time no one knows how much he can do, and the powers he possesses for toiling as well as enduring, until he has tried his powers and fathomed his resources. Let his aim, therefore, be a high one, and his confidence and hopefulness unshaken, for *much* is only accomplished by those who dare much and strive for much.'

D.— *Under what Special Conditions can a Woman undergo the Study of Dentistry?*

I have already mentioned that not all women are qualified to study dentistry, and will now endeavor to point out the requirements necessary for such study.

In the land of my birth, that staunch representation of conservatism, there is no chance given to women to study dentistry. The only university admitting females as students is that of Geneva, and there the female students are principally Russian ladies. A number of them, as I have learned, do not appear especially ladylike. Victoria Woodhull, Dr. Mary Walker, and that class of "strong-minded" women, would glory in seeing them walk through the streets, clad in tight trousers, with a student's cap on their heads, a cigar in mouth, flourishing a cane or a riding-whip in their right hand, and if reports are not exaggerated, occasionally joining male students in their nightly debauches and bacchanalian feasts. Since we do not yet have colleges and universities for the exclusive use of women, they are compelled to study in colleges, in which they are greatly outnumbered by male students, glad not to be ejected altogether. This being an undeniable fact, a woman, whether coming from Europe or being an American, must be without ties and without hopes; she must not be interested in men at all. Consequently she is not allowed to "flirt." This word is idiomatically English and has no representative in the German language.

Whoever of my sisters wants to enter upon the study of dentistry must be of most pure thoughts, which will produce pure words and lead to pure deeds. So much the more this will be the condition for those who "break the ice," who are the first adepts of the art.

A woman studying dentistry must abandon all thoughts of getting married. If she should marry, it would likely be to her repentance. The consequences of marriage would temporarily prevent her from performing the duties of her profession.

As concerns the place where a woman studies—the college—it had to be considered a "sanctuary," her behavior must be in strict accordance with this. Neither taking nor allowing the slightest liberties must be established as a rule from which under no condition a deviation is to be allowed. Then the gentlemen-students will have some respect for their female "commititones," and bear them, and not object to their joining in the lectures and demonstrations. In this way the faults found with us because of the very fact that we apply ourselves to the study of the professions I hope will be overcome, and the public in general will gradually acquire a more just opinion about the matter, and women hitherto jeered at and slighted for this attempt may yet be appreciated for trying to be useful in their way as far as their strength allows.

### *E.—What would be the Character of a Woman's Dental Practice?*

It is a matter of fact that out of ten individuals calling for the services of a dentist, from eight to nine are women and children. This statement being admitted, it is evident that although confined to this class of patients, there is a field sufficiently wide for woman's labor, and this in her proper sphere. Certainly almost any woman would greatly prefer to be treated by a woman, especially if she is in delicate circumstances. Moreover, nature has endowed woman with much more delicacy and tenderness of feeling, and has put into her heart more sympathy with the suffering of mankind than she gave to man.

I mentioned that among ten individuals applying for aid at a dentist's there were from eight to nine women and children. I will not search the cause why woman's teeth are liable to decay, I merely state the fact that women in delicate circumstances are more exposed to suffer from their teeth than others. Operations at this time, especially in the earlier stages of it, often resulting in sad consequences to the sufferer, a dentist ought to be careful to inquire about the state of the woman. This is a delicate point, especially for a young male dentist. He may seem too inquisitive and offend the patient. However as regards a lady dentist there is no harm in such inquiry. Out of a kind of bashfulness on both sides, the patient's, as well as the operator's, certain questions occasionally may not be made, and the sad consequences have to be borne. The statement has been made that occasionally unworthy members of the profession have taken liberties with ladies, who entrusted themselves to their care. There is no such thing to be apprehended with a lady dentist.

One German lady, Mrs. Henriette Hirschfeld, has been studying dentistry in Pennsylvania College, in Philadelphia, from 1867—1869. She is practising dentistry now, since September, 1869, in Berlin. Her success is encouraging to the sex. She counts among her patients even members of the imperial family. She is highly esteemed, enjoys the best reputation, and is prospering in every respect.

Empirism is the best judge of facts. Hence my hope that female dentists have a wide field of labor before them, not only in Germany but in many other countries, and may justly hope to meet with success.

### *Conclusion.*

They say that woman, for the physical strength denied to her by nature, is endowed with more perserverance and steadiness, and the deficiency of active force is made good by quickness of perception, greater elasticity and passive resistance. If this be so, and if some of these qualities may be found in me, I am sure I will make use of them to the best of my abililty. I shall proceed in the study of my profession and try to do honor, as well to my professors, to whom I owe the greatest thanks for their kindness so frequently shown to me, as to my sex, to whom I want to try to prove that a woman has a right to provide for her own wants.

P. S.—Since I wrote the above, I find in a German periodical of high standing — for 1872, No. 32 — an article on ladies studying at the University of Zurich. Von Bishoff, professor of anatomy and physiology, is opposed to their studying medicine, and declares that he never would allow any woman to attend his lectures. But another professor of the same university, member of the academic senate, has published in a daily paper—"*Allgamaina Zeitung*"—his views on the subject, and is rather in favor of the question.

The first two Russian ladies entered the university in 1864. Of twenty-five ladies having gone through a course of medical study, three left the university with a diploma, seven without being graduated. The number of female students who came in the winter of 1871-72 was from 19 to 31, and in the summer of 1872 from 31 to 63, of whom there were 54 Russian ladies. Foreigners are admitted without the *testimonium maturitatis*. Number of students of the University of Zurich was 208. Two ladies who have been graduated at Zurich also passed an examination in Russia and were permitted to practice medicine. They have located in Petersburgh, and enjoy a high reputation and have there an ample practice.

Not one word having been said of improper conduct of these ladies, I consider it my duty to take back what I have said about it, and am glad to do so for the sake of my sex.

